# Harnessing Machine Learning to Revolutionize Electrochemical Detection of Vitamin E Acetate in E‑Liquids

**DOI:** 10.1021/acsomega.5c02363

**Published:** 2025-06-13

**Authors:** Emine Sezer, Emre Dokuzparmak, Hilal Özçelik, Esra Yaşar, Tarık Kaya, Timuçin Güner, Sinan Akgöl

**Affiliations:** † Department of Computer Engineering, Faculty of Computer and Information Science, 37509 Ege University, Bornova, Izmir 35100, Turkey; ‡ Department of Bioengineering, Faculty of Engineering, Ege University, Bornova, Izmir 35100, Turkey; § Department of Biochemistry, Faculty of Science, Ege University, Bornova, Izmir 35100, Turkey; ∥ Sabancı University Nanotechnology Research and Application Center (SUNUM), Tuzla, Istanbul 34956, Turkey

## Abstract

This study presents the development of a novel molecularly imprinted electrochemical sensor for the sensitive and selective detection of vitamin E acetate (VEA) in e-cigarette liquids, a critical step in addressing the rising public health concern of e-cigarette, or vaping, product use-associated lung injury. VEA-imprinted polymeric nanoparticles, intended to serve as the recognition element on the sensor surface, were synthesized using surfactant-free emulsion polymerization. The synthesized polymer was characterized using Fourier Transformed Infrared Spectroscopy, scanning electron microscope, and zeta potential analyses. The sensor, fabricated using VEA-imprinted poly­(HMA-*co*-PA)/Nafion on screen-printed carbon electrodes, demonstrated a limit of quantification (LoQ) of 112.3 μg/mL (3.3× S/N) with a wide linear range extending to 3.0 mg/mL (10× S/N). While the sensor exhibited limitations in detecting VEA at concentrations below the LoQ, the integration of machine learning algorithms effectively mitigated these challenges. Machine learning models successfully classified the presence of VEA, even at subdetection limit concentrations, significantly enhancing the sensor’s analytical capabilities. Rigorous testing on real-world e-cigarette liquid samples yielded high recovery rates (96.83% ± 2.79–102.56% ± 3.84), validating the sensor’s accuracy and selectivity in complex matrices. This research not only establishes a promising platform for the rapid and sensitive detection of VEA in e-cigarette liquids but also underscores the transformative potential of integrating artificial intelligence with sensor technologies for addressing critical public health challenges.

## Graphical abstract

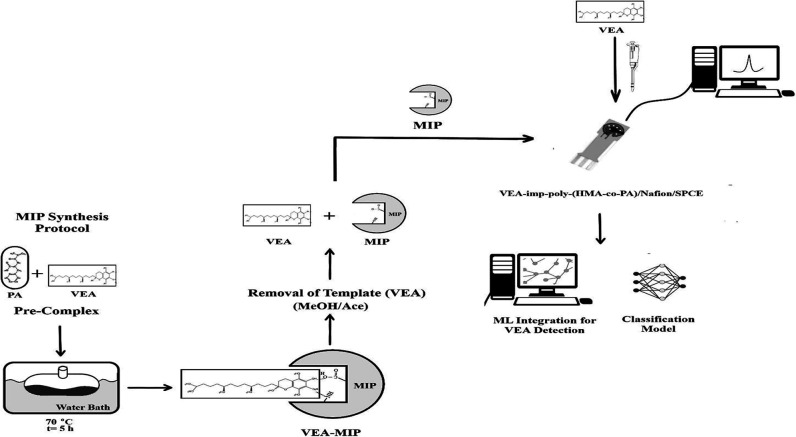

## 1 Introduction

Electronic cigarette (e-cigarette) devices are designed to simulate the experience of traditional smoking and deliver nicotine-containing aerosols. These devices comprise components such as a battery, heater, atomizer, e-liquid cartridge, and mouthpiece, each of which has the potential to adversely affect human health.^1,2^ Studies have identified over 113 distinct chemical components in e-cigarette cartridges and aerosols, with approximately 70 of these compounds remaining unidentified.^2–4^ Consequently, the identification of chemicals present in e-liquids and aerosols is crucial for a comprehensive assessment of the negative health impacts associated with e-cigarette use.

Mounting evidence suggests a link between e-cigarette use and lung damage, including respiratory failure. A recent outbreak of e-cigarette, or vaping, product use-associated lung injury (EVALI), as defined by the Centers for Disease Control and Prevention (CDC), underscored this concern and prompted urgent investigation. Vitamin E acetate (α-tocopherol acetate) (VEA), a component found in some e-cigarette liquids, has been implicated as a significant contributing factor in many EVALI cases.^5,6^

VEA is a fat-soluble vitamin recognized for its antioxidant properties and its role in mitigating oxidative stress.^7^ As an esterified form of vitamin E, VEA is commonly used in pharmaceuticals, cosmetics, and dietary supplements.^8^ In e-cigarette liquids, VEA is often added as a humectant and to enhance the delivery of flavors and nicotine.^6^ However, inhalation of VEA in aerosol form can pose serious health risks.^9^

Pulmonary surfactant (PS), a fluid lining the lung alveoli, plays a critical role in respiration. PS acts at the air–liquid interface to reduce alveolar surface tension, facilitating lung inflation under a pressure of approximately 133 pascal (Pa). Phospholipids, primarily dipalmitoylphosphatidylcholine (DPPC), constitute the major component (90%) of PS, along with other lipid components such as unsaturated phosphatidylcholines (PCs) and phosphatidylglycerols (PGs).^9^

Inhaled VEA can accumulate in the alveolar membrane, potentially inducing inflammation and lung damage.^10^ Studies employing model systems have investigated the mechanisms of VEA-induced lung damage.^11,12^ Models using DPPC alone have demonstrated that increasing VEA concentrations negatively affect membrane fluidity and compressibility. Similar adverse effects on membrane fluidity and compressibility were observed in models using a mixture of the four main PS components: phospholipids (90%), neutral lipids (8%), proteins (2%), and glycolipids.^9^ These findings suggest that VEA increases PS fluidity, potentially leading to alveolar collapse and respiratory symptoms such as shortness of breath and lung inflammation.

A comprehensive review of the existing literature has revealed an absence of studies pertaining to the development of a sensor system specifically designed for the detection of VEA in electronic cigarettes. Additionally, traditional methods for detecting chemicals in e-cigarette liquids, such as polarographic analysis, liquid chromatography, UV spectrophotometry, gas chromatography, and capillary electrophoresis,^12^ often involve lengthy analysis times, complex sample preparation, and expensive equipment. Electrochemical sensors offer a compelling alternative for rapid, cost-effective, and sensitive detection of vitamins and other chemicals.^13^ Their high sensitivity, rapid response times, and potential for miniaturization have led to their widespread use in biological and environmental analyses.^14^ These sensor systems enable reliable and rapid detection of harmful substances in biological fluids, making them valuable tools in public health monitoring.^15^

Molecularly imprinted polymers (MIPs), characterized by their inherent capacity for specific molecular recognition, have garnered significant attention in recent years as a promising technology for the development of sensitive and selective sensors for target analytes.^16–19^ Molecularly imprinted electrochemical sensors (MIECS), which employ MIPs as recognition elements, offer a low-cost, rapid, and reliable detection method applicable even in complex matrices.^20,21^ Integration with disposable electrodes, such as screen-printed carbon electrodes (SPCEs), further expands their utility, particularly in electrochemical biosensing.^22^

Despite the widespread adoption of electrochemical sensors across diverse analytical domains, their performance under nonlinear conditions and low analyte concentrations remains a persistent challenge. Conventional calibration protocols and signal analysis techniques frequently exhibit limited capacity in resolving weak, noisy, or ambiguous signals—particularly in the quantification of analytes such as VEA present at trace levels, below conventional limits of detection. In light of these limitations, machine learning (ML) has emerged as a robust and scalable paradigm, offering data-driven strategies for both sensor optimization and interpretive enhancement.^23–25^ By systematically extracting latent patterns from historical sensor outputs, ML algorithms enable the precise characterization of signal dynamics, drift compensation, and classification of analyte presence even within chemically complex matrices.^26,27^ These capabilities collectively facilitate enhanced analytical resolution, improved selectivity, and elevated signal-to-noise performance, particularly under nonideal measurement conditions.^28^

Beyond signal refinement, ML methodologies contribute to the rational design and optimization of sensor materials and configurations by enabling predictive modeling of structure–performance relationships. This computational foresight not only expedites sensor development cycles but also fosters the engineering of customized sensing interfaces with tailored selectivity and sensitivity profiles.^29^ In conjunction with dimensionality reduction and feature extraction techniques—such as principal component analysis (PCA) and wavelet transform methods—ML frameworks augment the interpretability of complex electrochemical signals and support reliable analyte discrimination in multidimensional data spaces.^30,31^ Moreover, data-driven approaches have demonstrated efficacy in addressing instrumental drift, calibration bias, and long-term signal instability, thereby enhancing the robustness and reproducibility of electrochemical platforms.^32^

Within the scope of supervised ML applications in electrochemical sensor analytics, several algorithmic frameworks have demonstrated consistent effectiveness across a wide range of data complexities. Techniques such as support vector machines (SVM), random forest ensembles, artificial neural networks (ANNs), logistic regression, and gradient-boosted decision trees (e.g., XGBoost) have been particularly prominent due to their capacity to model intricate, high-dimensional, and nonlinear relationships with robust generalization performance.^33–41^ The selection of an appropriate algorithm is inherently dependent on the structure and statistical properties of the data set, including its dimensionality, variance distribution, and noise characteristics. In this context, kernel-based models and ensemble learning approaches have shown distinct advantages, particularly in handling high-variance data sets or those involving complex class boundaries. Their ability to capture both linear and nonlinear feature interactions renders them highly suitable for the classification tasks frequently encountered in chemometric and biosensing applications.

In this study, a novel and integrated sensing strategy was developed for the selective and sensitive detection of VEA in e-cigarette liquids, addressing a critical gap in current analytical methodologies. The platform combines MIP-based nanostructures synthesized via surfactant-free emulsion polymerization with an electrochemical detection system built upon SPCEs modified by a poly­(HMA-*co*-PA)/Nafion composite. Extensive physicochemical and electroanalytical characterization confirmed the structural fidelity, binding specificity, and electrochemical responsiveness of the fabricated sensor. To overcome the intrinsic limitations of conventional sensors—particularly under nonlinear response conditions or subquantification levels—supervised ML models were systematically integrated into the analytical workflow. These models not only enhanced the interpretive capacity of the electrochemical data by distinguishing subtle signal variations but also enabled reliable classification of VEA presence in chemically complex and real-world matrices. Collectively, the interdisciplinary integration of molecular imprinting, electrochemical transduction, and artificial intelligence exemplifies a forward-looking and robust platform, with significant implications for rapid toxicant detection, public health surveillance, and the future design of intelligent sensor systems.

## 2 Experimental Design and Machine Learning Integration

This section details the synthesis, characterization, electrochemical measurements, and ML-based optimization strategy employed for the development of MIECS for VEA detection.

### 2.1 Materials and Reagents

VEA was procured from Sigma-Aldrich. The following reagents were used in the polymerization process: poly­(vinyl alcohol) (PVA) (≥99.0%), potassium persulfate (KPS) (≥99.0%), 2-hydroxyethyl methacrylate (HMA) (≥99.0%), ethylene glycol dimethacrylate (EGDMA) (≥99.0%), dimethyl sulfoxide (DMSO) (≥99.9%), methanol (MeOH) (≥99.9%), ethanol (EtOH) (≥99.5%), and acetic acid (AA) (≥99.7%), methacryloylamido phenylalanine (PA). For the electrochemical measurements, potassium chloride (KCl), potassium hexacyanoferrate­(III) (K_3_[Fe­(CN)_6_]), potassium hexacyanoferrate­(II) trihydrate (K_4_[Fe­(CN)_6_]·3H_2_O), and potassium dihydrogen phosphate (KH_2_PO_4_) were employed. All chemicals used in the study were obtained from Sigma-Aldrich (St. Louis, MO, USA). An 5.0% (w/v) Nafion 117 solution (methanol/isopropyl alcohol) was sourced from Chemours (Wilmington, DE, USA). All chemicals were of analytical grade and used as received without further purification.

### 2.2 Instrumentation

All electrochemical analyses were performed using a Potentiostat/Galvanostat (μStat 400-Metrohm, Herisau, Switzerland) interfaced with a SPCE system. The SPCE (DropSens, Llanera, Spain) was modified with a VEA-imp-poly­(HMA-*co*-PA)/Nafion composite, functioning as the working electrode. The SPCE consisted of a carbon working electrode (4 mm diameter), a silver pseudoreference electrode, and a carbon counter electrode. Auxiliary equipment included a shaking water bath (Memmert, Schwabach, Germany; WiseBath WSB-30), Centurion Scientific Ltd., St Neots, UK and Beckman Coulter Avanti J-E, Beckman Coulter, Inc., Brea, CA, USA.

### 2.3 Synthesis of VEA-imp-poly­(HMA-*co*-PA) (MIP) and Nonimprinted Polymer (NIP)

VEA-imprinted polymeric nanoparticles, intended to serve as the recognition element on the sensor surface, were synthesized using surfactant-free emulsion polymerization. A precomplex was prepared by mixing 25 mg of VEA with 25 mL of PA in DMSO and stirring for 2 h. Separately, 0.5 g of PVA was dissolved in 45 mL of deionized water under magnetic stirring. HMA (0.6 mL) and EGDMA (0.3 mL) were then added to this solution, which was subsequently combined with the precomplex solution in a polymerization reactor. The mixture was purged with nitrogen gas for 10 min to remove dissolved oxygen before initiating polymerization with 0.02 g of KPS dissolved in 45 mL of deionized water. Polymerization was carried out at 70 °C for 5 h under constant stirring. The resulting polymers included three MIPs with varying VEA/PA molar ratios (1:1, 1:2, and 1:3) and one NIP synthesized without the template molecule. Following polymerization, the MIPs and NIP nanoparticles were centrifuged at 14,100 rpm for 20 min and washed three times with a 50% ethanol–water mixture to remove unreacted monomers and residual impurities. The polymeric nanoparticles were then dried in an oven at 37 °C for 12 h. Subsequently, to extract the template molecule, the particles were extensively washed with a methanol/acetic acid (9:1, v/v) solution, which was experimentally determined to be optimal for template removal, and then dried under vacuum at 40 °C overnight. The methanol/acetic acid (9:1, v/v) solution was experimentally determined to be optimal. This solution is commonly used in the literature for the removal of template molecules from MIPs. Methanol facilitates the dissolution of the template from the polymer matrix, while acetic acid disrupts ionic and hydrogen bonds, collectively enhancing desorption efficiency.^42–44^

### 2.4 Fabrication of VEA-imp-poly­(HMA-*co*-PA)/Nafion/SPCE

The SPCE modification involved preparing a suspension of VEA-imp-poly­(HMA-*co*-PA) in a 0.05% Nafion solution (prepared by diluting the 5% stock solution with deionized water) to achieve a final concentration of 1.0 mg/mL. A 15 μL aliquot of this composite solution was drop-cast onto the SPCE surface and allowed to dry at room temperature (22 ± 2 °C) in the dark for 2 h. Afterward, the modified electrodes were stored at 4 °C for subsequent analyses.

### 2.5 Electrochemical Measurements

Electrochemical measurements were conducted using differential pulse voltammetry (DPV) and cyclic voltammetry (CV) within a potential range of −0.20 to +0.80 V in 10 mM phosphate buffer saline (PBS, pH 7.2) containing 5.0 mM [Fe­(CN)_6_]^4–/3–^ as the redox probe. All measurements were performed using a three-electrode configuration: the modified SPCE as the working electrode, a silver pseudoreference electrode on the SPCE, and the carbon counter electrode on the SPCE. Preliminary CV scans were performed at varying scan rates (10–100 mV/s) to determine the optimal scan rate of 60 mV/s, which provided the highest peak current and well-defined peak shape. Further details regarding the instrumentation and analytical procedures can be found in our previous publications.^45–47^

### 2.6 Machine Learning-Assisted Optimization and Classification

Accurate identification of VEA at trace levels necessitates the deployment of robust classification models capable of minimizing false negatives—a critical consideration given the compound’s established association with EVALI.^48^ To address this challenge, five supervised machine learning algorithms—including SVM, random forest, neural networks (NNs), logistic regression, and XGBoost—were trained using electrochemical current responses acquired across a range of applied voltages. Each sample was labeled with a binary class indicator denoting the presence or absence of VEA.

The data set was preprocessed to eliminate redundancy and reduce the risk of overfitting through standardization and stratified sampling. Hyperparameter tuning was performed individually for each algorithm to ensure optimal performance. Evaluation extended beyond overall accuracy and incorporated recall and F1-score metrics, which emphasize true positive identification while accounting for the balance between sensitivity and precision.^49^

### 2.7 Integration of ML Insights into Experimental Workflow

Rather than functioning as isolated computational instruments, the trained ML models were strategically integrated into the experimental workflow to refine signal interpretation and enhance resolution, particularly within the low-concentration detection regime. These models enabled characterization of the sensor’s nonlinear electrochemical behavior at subquantification thresholds—an area where conventional calibration methods are often insufficient.

By using applied voltage and the corresponding current as input features, the ML models predicted the likelihood of VEA presence with high interpretive confidence. This predictive layer supported the resolution of ambiguous signals and guided the refinement of experimental validation strategies. As such, the incorporation of ML not only complemented empirical observation but also exemplified a synergistic interplay between data-driven inference and sensor-based experimentation, ultimately contributing to a more nuanced and reliable detection process.

## 3 Results and Discussion

### 3.1 Synthesis and Characterization of VEA-imp-poly­(HMA-*co*-PA)

Polymeric nanoparticles imprinted with VEA, designed to function as the recognition element on the sensor surface, were synthesized through surfactant-free emulsion polymerization. As illustrated in Figure 1, the interaction between VEA and PA in the precomplex is predominantly driven by hydrophobic interactions originating from the phenylalanine amino acid, with additional binding affinities facilitated by other secondary interactions. Upon polymerization, the removal of VEA from the structure enabled the synthesis of a polymer matrix containing cavities specific to VEA.

**Figure 1:**
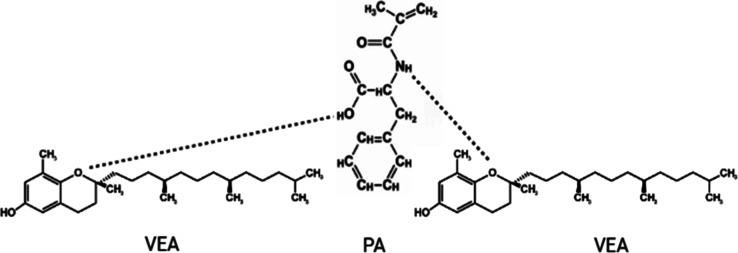
Schematic representation of the pre-complex formation between PA and VEA.

#### 3.1.1 Fourier Transform Infrared Spectroscopy (FTIR) Analysis

The FTIR analysis of VEA-imprinted poly­(HMA-*co*-PA) confirms the successful incorporation of the template molecule (VEA) within the polymer matrix. Key spectral differences between the imprinted and nonimprinted polymers indicate specific interactions, validating the molecular imprinting process and its potential for selective sensing applications.

The FTIR spectrum analysis reveals significant structural differences between the P­(HMA) and P­(HMA-*co*-PA) polymers. In the region of 3300–3500 cm^–1^, broad absorption bands corresponding to the hydroxyl (−OH) groups of HMA and the amide (−NH) groups of PA are observed. A slight shift in this region for the imprinted polymer suggests the formation of hydrogen bonds with the template molecule, vitamin E. The aliphatic C–H stretching vibrations in the range of 2900–3000 cm^–1^ appear in both spectra, though intensity variations indicate differences in polymerization and cross-linking as shown in Figure 2. Distinct peaks in the 1700–1750 cm^–1^ region correspond to the carbonyl (C=O) stretching vibrations of HMA’s ester groups and PA’s amide groups.^50^ Notable shifts and intensity variations in this region for the imprinted polymer provide strong evidence of interactions between vitamin E and the polymer matrix, confirming the success of the molecular imprinting process. The 1500–1650 cm^–1^ region exhibits characteristic absorption bands associated with C=N stretching and NH bending vibrations of PA’s amide functionalities, with observable differences between the imprinted and nonimprinted polymers. Furthermore, the 1000–1300 cm^–1^ range features stretching vibrations of C–O–C bonds from HMA’s ester structure and C–N bonds from PA, both of which show distinct spectral features. The fingerprint region (500–900 cm^–1^) reflects the specific structural characteristics of the polymer, where noticeable spectral variations in the imprinted polymer further support the structural modifications induced by the presence of vitamin E. Overall, the FTIR data confirm the successful molecular imprinting process and demonstrate specific interactions between the polymer matrix and vitamin E, validating the system’s structural integrity and functionality.

**Figure 2:**
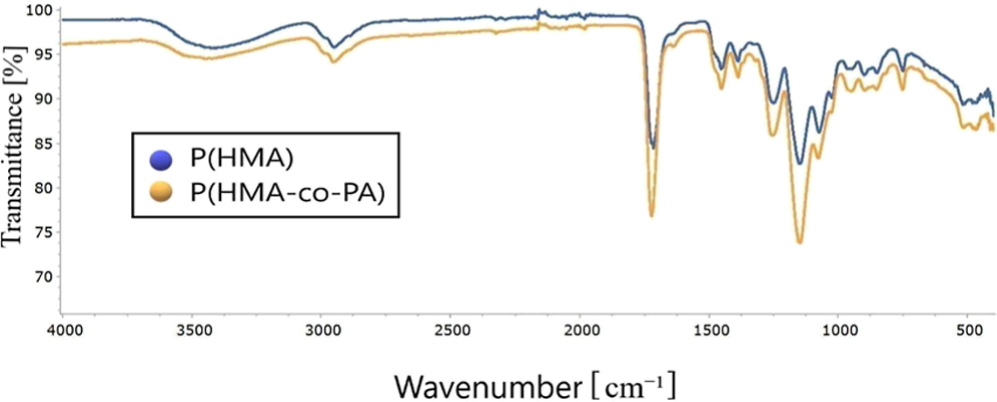
Comparative FTIR spectra of P­(HMA) and P­(HMA-*co*-PA) polymeric nanoparticles.

#### 3.1.2 Scanning Electron Microscope (SEM) Analysis

According to the SEM analysis presented in Figure 3, the VEA-imprinted poly­(HMA-*co*-PA) nanoparticles predominantly exhibit a spherical morphology with a relatively rough and porous surface structure. This morphology is highly advantageous for molecular imprinting applications, as the spherical shape provides a high surface-to-volume ratio, while the rough surface offers a greater number of accessible binding cavities. These structural features enhance the performance of the polymer by enabling stronger and more specific interactions with the template molecule.

**Figure 3:**
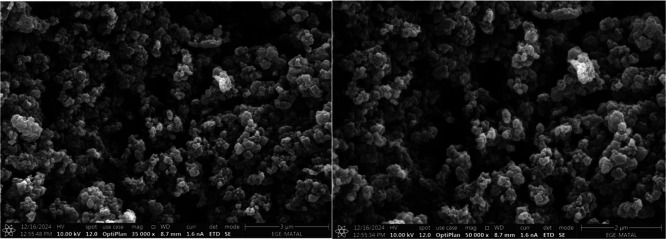
SEM images of the MIPs: VEA-imp-poly­(HMA-*co*-PA).

#### 3.1.3 Zeta Size and Potential Analysis

The zeta potential analysis of VEA-imp-poly­(HMA-*co*-PA) polymeric nanoparticles provides valuable insights into their surface charge and colloidal stability (Figure S1). The average zeta potential was measured as −2.36 mV, indicating a near-neutral surface charge, which may be beneficial for minimizing nonspecific interactions in biological applications. A single dominant peak (peak 1: −2.36 mV, 100%) in the zeta potential distribution suggests a homogeneous nanoparticle population with consistent surface charge characteristics. The zeta potential deviation was recorded as 7.12 mV, indicating a relatively narrow charge distribution. Additionally, the conductivity was measured at 3.97 mS/cm, and the result quality was rated as “good”, confirming the reliability of the measurement. These findings suggest that the synthesized nanoparticles exhibit a stable and uniform charge profile, which can be further optimized for enhanced colloidal stability and potential biomedical applications.

The dynamic light scattering (DLS) analysis of VEA-imp-poly­(HMA-*co*-PA) polymeric nanoparticles, as presented in Figure S2, indicates the successful synthesis of nanoparticles. The *Z*-average value was determined to be 759.2 nm, providing essential information about the hydrodynamic diameter of the polymeric system. A single dominant peak was observed in the size distribution (peak 1:177.9 nm, 100%), suggesting the formation of a homogeneous nanoparticle population within a specific size range. The polydispersity index (PdI) was measured as 0.726, indicating a well-distributed nanoparticle system. These findings demonstrate that the synthesized nanoparticles fall within the desired size range and may be suitable for potential biomedical applications (Figure S2).

### 3.2 Optimization of Binding Conditions

A calibration curve was generated to establish the correlation between absorbance values and the concentrations of VEA solutions, serving as a reference for accurately determining VEA concentrations in subsequent experiments (Figure S3).

The calibration process involved preparing aqueous solutions with five different concentrations of VEA, ranging from 0.1 to 1 mg/mL. Using a UV–vis spectrophotometer, the absorbance of each solution was measured at a wavelength of 286 nm. The calibration curve was then constructed by plotting absorbance values against the respective VEA concentrations (mg/mL) (*R* ^2^ = 0.9884). This calibration curve provides a quantitative relationship between absorbance and VEA concentration, offering a reliable reference for accurately measuring concentrations in spectrophotometric analyses.

#### 3.2.1 Effect of pH on VEA Binding

To evaluate the effect of pH on VEA binding, 10 mM buffer solutions were prepared, and experiments were conducted at pH levels of 5.0, 6.0, 7.0, 8.0, 9.0, and 10.0 to examine the working conditions at different pH values. Binding studies were performed at room temperature with a total volume of 1 mL. A solution containing VEA and 6 mg of polymer was prepared with an initial VEA concentration of 1.0 mg/mL, and allowed to adsorb for 2 h using the continuous binding method. The amount of VEA adsorbed by the polymer at each pH was calculated using eq 1.

1 $$Q=\frac{C_{i}-C_{f}}{m}\times V$$

In this eq 1, *Q* represents the amount of VEA bounded per unit mass of polymer (mg/g), while “*c*_i_” and “*c*_f_” denote the initial concentration of VEA in the solution and its concentration in the aqueous phase after a specified time, respectively. “*V*” indicates the volume of the aqueous phase (mL), and “*m*” refers to the mass of the polymer used (mg). The calculated values were subsequently plotted to identify the optimal pH conditions for efficient binding.

Figure 4 demonstrates a notable increase in the binding capacity of VEA by the MIP as the pH increased from 5.0 to 7.0. The highest VEA binding value was observed as 86.538 mg/g in the pH 7.0 buffer. However, a significant decrease in the binding capacity was observed at pH 8.0, indicating that the interaction between VEA and the MIP was less favorable under these conditions. This reduction could be attributed to the full deprotonation of the carboxylic acid groups on the polymer surface, resulting in increased electrostatic repulsion and a weakened binding affinity for VEA.

**Figure 4:**
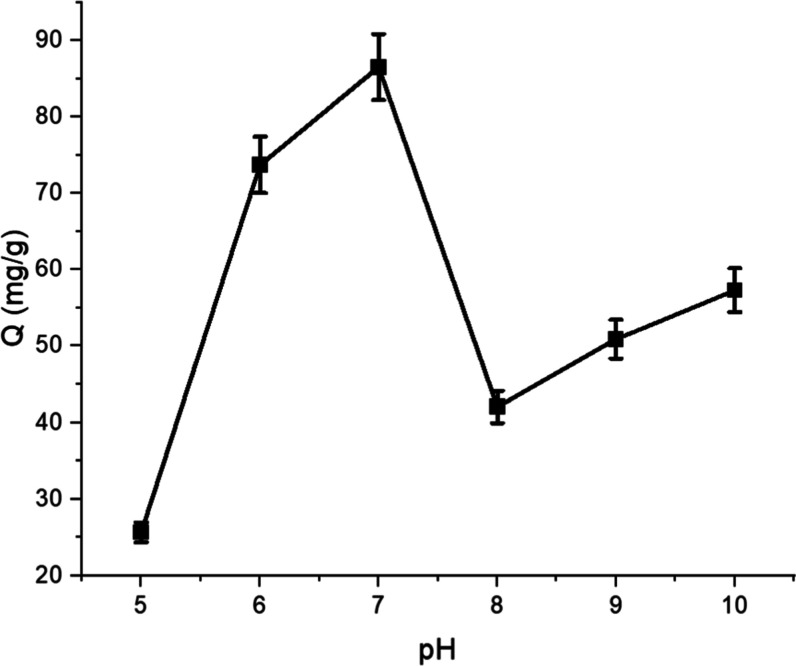
pH effect on VEA binding to VEA-imp-poly­(HMA-*co*-PA) (*C*_VEA_: 1.0 mg/mL, *T*: room temperature, *t*_binding_: 2 h, pH 5.0 sodium acetate buffer, pH 6.0–7.0–8.0 phosphate buffer, pH 9.0 Tris buffer, pH 10.0 borate buffer).

At pH values beyond 8.0, such as in the pH 9.0 buffer, the binding capacity stabilized at a lower level, suggesting that while some noncovalent interactions (e.g., hydrogen bonding or hydrophobic interactions) still contribute to binding, the overall affinity of the MIP for VEA is diminished under these alkaline conditions. These results highlight the pH-sensitive nature of the MIP and its optimal performance at neutral pH levels.

#### 3.2.2 Effect of Initial Concentration on VEA Binding

To investigate the effect of initial concentration on VEA binding, binding media were prepared with initial concentrations of VEA ranging from 0.1 to 1 mg/mL, with the final volume adjusted to 1 mL using a pH 7.0 sodium phosphate buffer solution. The desired concentration was achieved by accurately pipetting a 1 mg/mL VEA/solvent stock solution, followed by the addition of 6 mg of polymer to each solution. The experiments were conducted at room temperature. After the binding phase, the samples were centrifuged at 14.000 rpm for 20 min, and VEA analysis was performed on the resulting supernatants. Control trials, which did not include the polymer, were carried out using the same procedure to establish baseline values for each concentration level. These control results were used to determine the initial concentration for each value in the experimental setup. The effect of the initial VEA concentration on its binding is shown in Figure 5.

**Figure 5:**
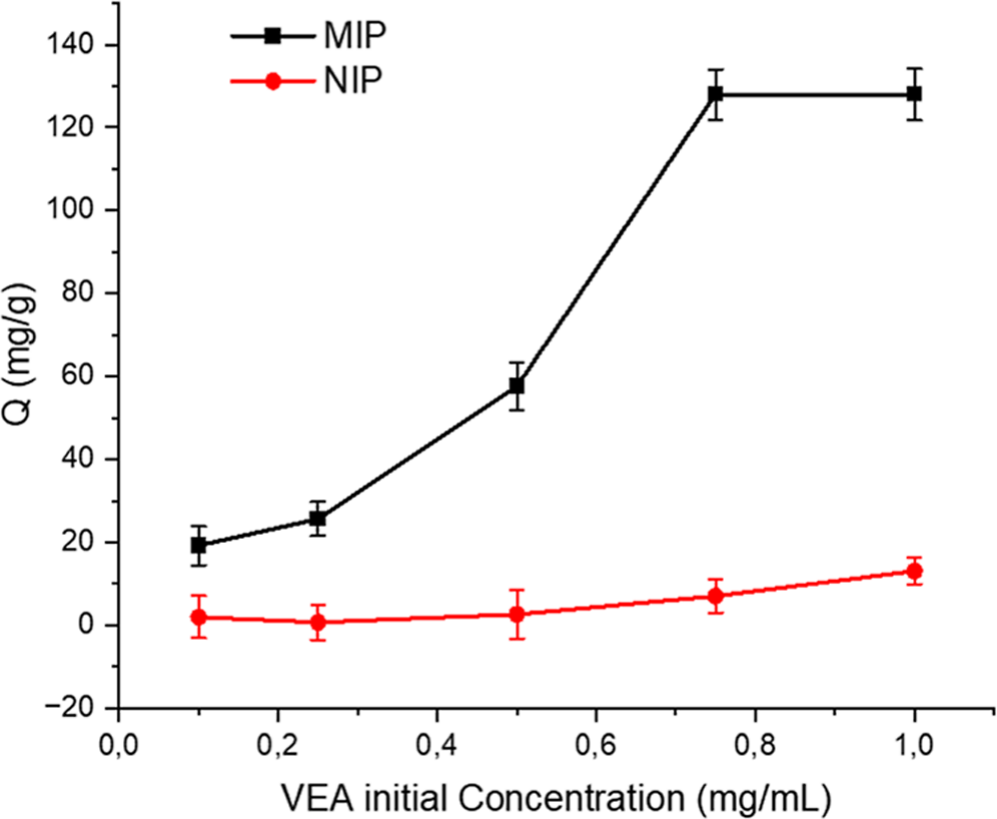
Effect of initial concentration on VEA binding (pH 7.0 sodium phosphate buffer, 25 °C, binding time: 2 h for MIP and NIP).

Figure 5 illustrates the effect of the initial concentration of VEA on the binding capacity of MIP and NIP. For MIP, a gradual increase in binding capacity was observed at lower initial concentrations (0.1–0.4 mg/mL), indicating that the specific binding sites on the polymer surface were not yet saturated and continued to interact effectively with the target molecules. As the concentration increased within the range of 0.4–0.8 mg/mL, a significant rise in binding capacity was noted, highlighting the strong selectivity and affinity of MIP for the target molecule. However, beyond 0.8 mg/mL, the binding capacity reached a plateau at its maximum value (approximately 120 mg/g), indicating that all specific binding sites on the polymer surface were fully occupied and further binding could not occur.

When the results of the NIP study are evaluated, the role of hydrophobic interactions in molecular recognition has been emphasized in imprinting studies, demonstrating that even in nonimprinted systems, molecular adsorption can occur through nonspecific interactions, particularly at higher analyte concentrations.^51^ NIPs can exhibit adsorption due to noncovalent interactions, including π–π stacking and hydrophobic forces.^52,53^ However, in molecular imprinting studies, minimizing nonspecific adsorption in NIPs is crucial to accurately differentiate the binding performance of NIP and MIP systems. Ideally, NIPs should exhibit minimal interaction with the target analyte, serving as a control to validate the selectivity and specificity of MIPs. Although a slight increase in Q values was observed in NIP experiments, this increase remained at a very low level. The selective recognition of VEA by MIP was confirmed to result from the specific binding sites formed within the polymer matrix.

NIPs serve as a “control” to evaluate the selectivity of the interactions between the synthesized MIPs and the template molecule, as these interactions are specific to MIPs and not to NIPs; compared to NIPs, MIPs exhibit better binding capacity and higher selectivity, and the calculated binding ratio between MIPs and NIPs is referred to as the imprinting factor (IF) (Equation 2).^54^

2 $$\alpha =Q_{MIP}/Q_{NIP}$$

Here, *Q*_MIP_ and *Q*_NIP_ represent the binding capacities in a monolayer polymer surface.

In this study, the calculated imprinting factor (IF) was found to be 8, indicating a high degree of specificity and successful imprinting.

### 3.3 Electrochemical Characterization

#### 3.3.1 Detection of the Optimum Scan Rate

The electrochemical behavior of the VEA-imp-poly­(HMA-*co*-PA)/Nafion/SPCE was examined in the potential range of −0.20 to +0.80 V using a 5.0 M Fe­(CN)_6_^3–^/^4–^, with scan rates (ν) between 10 and 100 mV/s (Figure 6A). The Fe­(CN)_6_^3–^/^4–^ system displayed a reversible single-electron oxidation process. It was also noted that both the oxidation and reduction peaks increased linearly across the scan rate range. This helped identify the scan rate at which the modified SPCE could function effectively without encountering diffusion limitations (Figure 6A). To achieve both rapid analysis, high electrochemical stability and high accuracy in the experiments, a scan rate of 60 mV/s was selected as the optimum scan rate.

**Figure 6:**
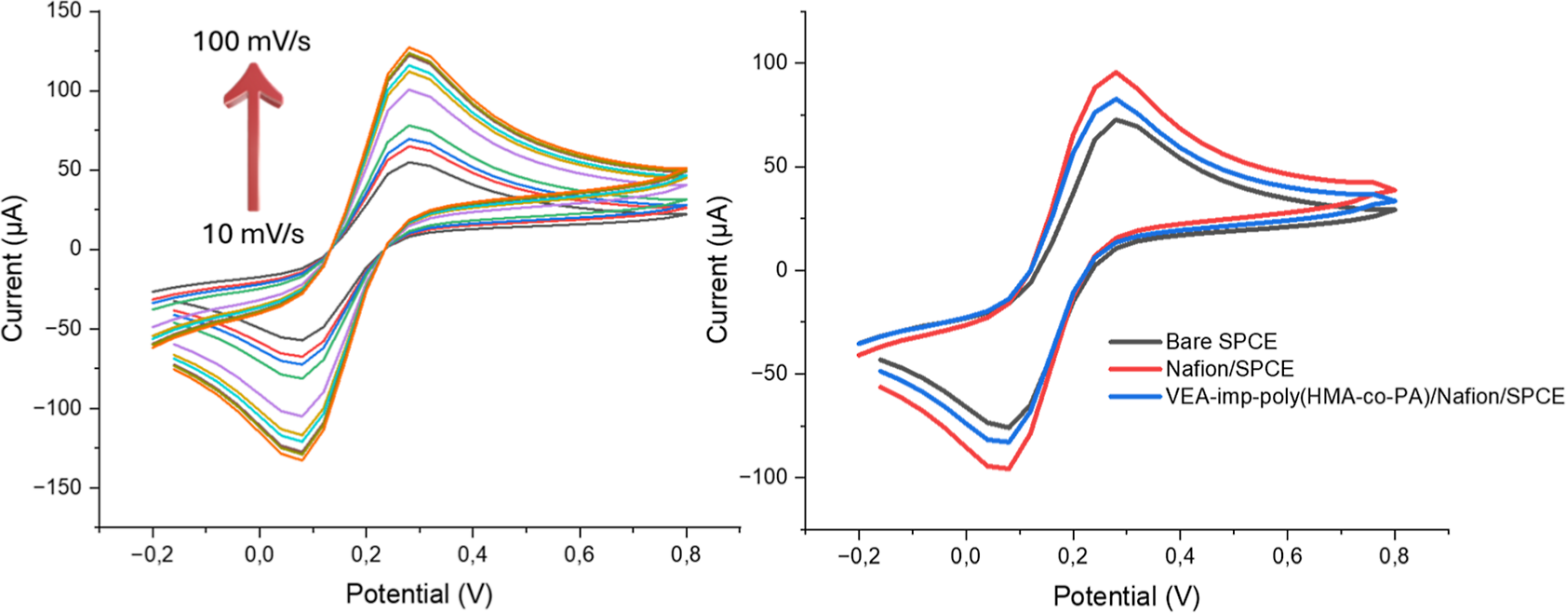
(A) Cyclic voltammograms obtained at scan rates ranging from 10 to 100 mV/s^–^, (B) CVs of the bare SPCE, Nafion/SPCE, and VEA-imp-poly­(HMA-*co*-PA)/Nafion/SPCE in a 5.0 mM Fe­(CN)_6_^3–^/^4–^.

#### 3.3.2 Fabrication of VEA-imp-poly­(HMA-*co*-PA)/Nafion/SPCE

The fabrication of the VEA-imp-poly­(HMA-*co*-PA)/Nafion/SPCE sensor involved performing CV on the bare SPCE electrode using a 5.0 mM Fe­(CN)_6_^3–^/^4–^. The potential range for the measurements was −0.2 to 0.8 V, with a scan rate of 60 mV/s. To enhance the electrode surface area and immobilize the synthesized nanomaterials onto the surface, CV measurements were carried out in the presence of 0.5% (w/v) Nafion polymer. The electrochemical performance of the SPCE electrode system was then assessed. Furthermore, the electrochemical behavior of the sensor was further analyzed after immobilizing both Nafion and 1.5 mg/mL of VEA-imp-poly­(HMA-*co*-PA) polymer onto the electrode surface (Figure 6B).

Figure 6B shows that when the bare SPCE electrode was modified with a Nafion film, a significant increase in current values was observed in the cyclic voltammograms. This increase is attributed to the enhanced electrode surface area from the Nafion polymer modification, as well as Nafion’s conductive properties, which improved electron transfer and resulted in a noticeable current increase.^55^ However, when the synthesized VEA-imp-poly­(HMA-*co*-PA) was added to the Nafion-modified SPCE surface, a decrease in current values was observed. This decrease is due to the nonconductive nature of the added polymer, which impedes electron flow at the electrode surface. Based on the obtained CV results, the electrochemical explanation of the sensor surface modification and sensor fabrication process has been provided and demonstrated.

#### 3.3.3 Effect of Nafion Concentration

Nafion is a widely used cation exchange polymer, characterized by its distinct structure, which includes a fluorocarbon backbone (polytetrafluoroethylene) and pendant ionic groups like SO_3_^–^ attached to side chains. This structure imparts Nafion with exceptional electrochemical and thermal properties, such as efficient proton transport, selective ion exchange, strong catalyst support, and chemical stability. These attributes contribute to Nafion’s resistance to chemical degradation, even under harsh conditions like high temperatures and exposure to strong oxidants.^56,57^

It has been reported in several studies that the coating of Nafion on the electrode surface leads to changes in the effective electrode area. In addition, as a cation-exchange polymer, Nafion has a direct impact on the conductivity of the system. Theoretically, when the electrode surface is modified with a polymer that influences conductivity, alterations in the surface area and corresponding changes in current response are to be expected.^58–60^

For this study, a film solution of VEA-imp-poly­(HMA-*co*-PA)/Nafion was prepared by mixing Nafion 117 solution with methanol concentrations ranging from 0.1% to 1% (w/v). A 15 μL drop of the methanol-containing solution was applied to the surface of each electrode surface, and the electrodes were left to dry for 2 h at room temperature before being stored at +4 °C for future use. DPV was conducted at a scan rate of 60 mV/s over a potential range of −0.20 to +0.60 V. The highest current response was observed with 0.5% Nafion (Figure 7). Higher concentrations of Nafion led to a decrease in current, likely due to diffusion limitations of Fe­(CN)_6_^3–^/^4–^ ions.

**Figure 7:**
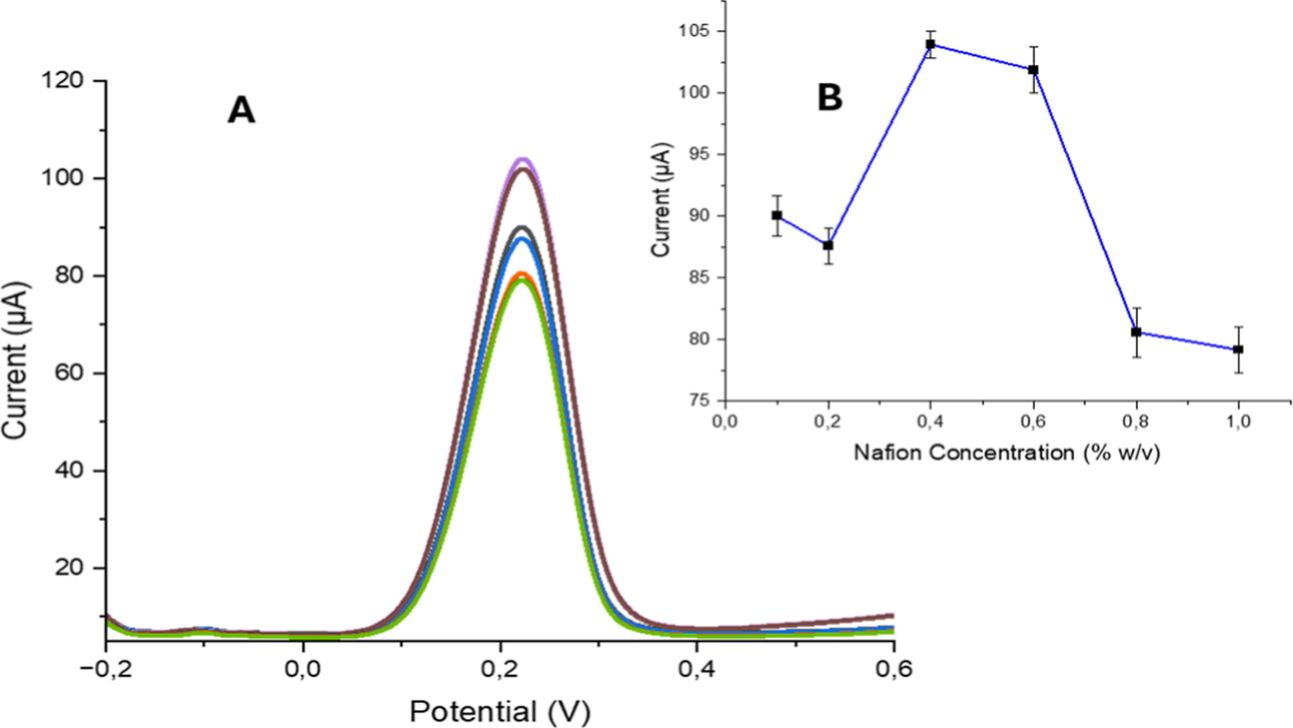
(A) DPV results (B) DPV peak values of 5 mM Fe­(CN)_6_^3–^/^4–^ were obtained for different Nafion concentrations (from 0.1% to 1% (w/v)).

Among the various concentrations tested, the highest current response was observed at 0.5% Nafion, as illustrated in Figure 7. However, at concentrations exceeding 0.5%, a decline in the current response was attributed to diffusion limitations of Fe­(CN)_6_^3–^/^4–^ ions through the thicker Nafion layer. These results align closely with those reported in the literature.^61^ The optimal Nafion concentration was determined to be 0.5% (w/v).

#### 3.3.4 Effect of Polymer Concentration

The VEA-imp-poly­(HMA-*co*-PA) polymer’s inherently low conductivity leads to a reduced overall conductivity of the electrode system when applied to the electrode surface. Therefore, it is crucial to determine the optimal polymer loading on the electrode to achieve the highest current values. Polymer concentrations ranging from 0.25 to 2.0 mg/mL of VEA-imp-poly­(HMA-*co*-PA), combined with 0.5% Nafion, were tested. As shown in Figure 8, a concentration of 1.5 mg/mL of VEA-imp-poly­(HMA-*co*-PA) was identified as the optimal value, offering an adequate active surface for accurate VEA measurement.

**Figure 8:**
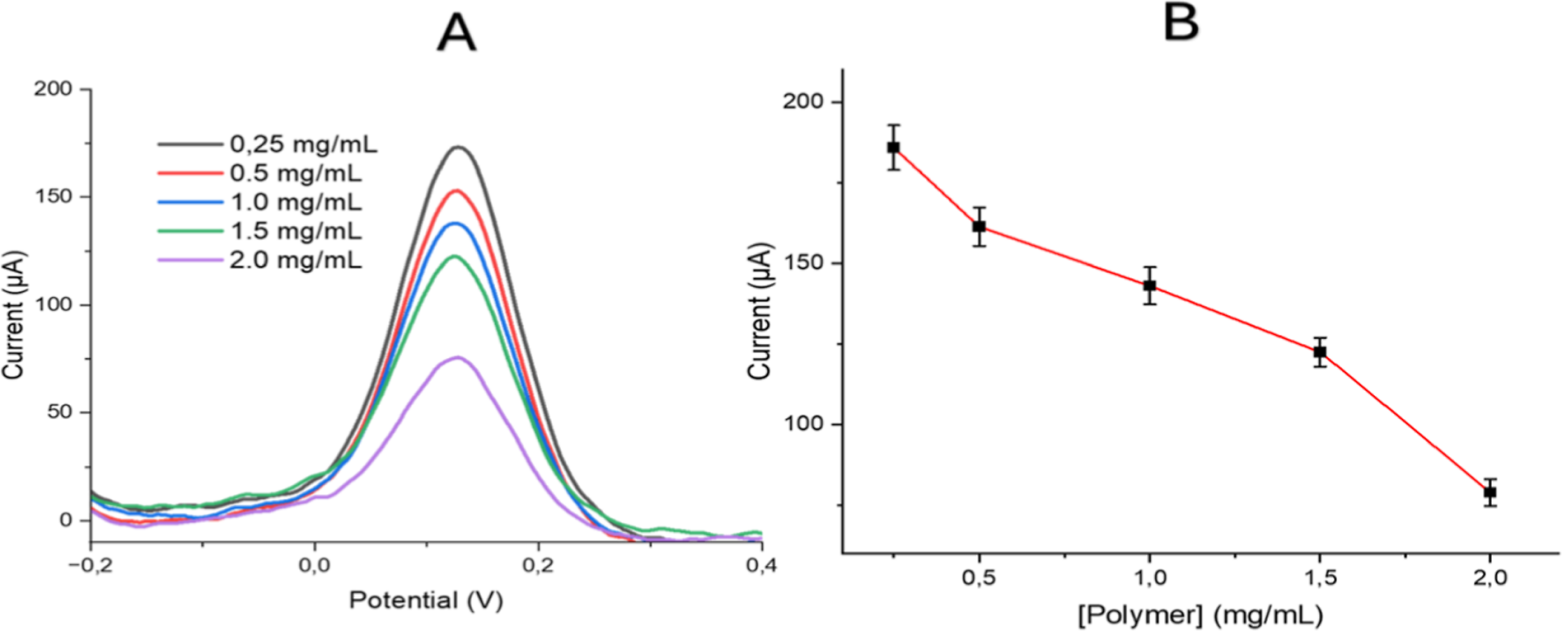
(A) DPV results (B) DPV peak values of VEA-imp-poly­(HMA-*co*-PA)/Nafion/SPCE in 10 mM Fe­(CN)_6_^3–/4–^ using varying concentration of VEA-imp-poly­(HMA-*co*-PA) (ranging from 0.25 mg/mL to 2 mg/mL).

Loading different concentrations of the polymer onto the SPCE surface altered the electrode surface and disrupted the diffusion equilibrium. This disrupted diffusion equilibrium resulted in a decrease in current values in the presence of a redox agent. Due to the significant reduction in diffusion at high polymer quantities, an optimal value was determined to achieve both maximum selectivity and maximum current values. Current values were observed to dramatically decrease at polymer concentrations higher than 1.5 mg/mL, thus establishing 1.5 mg/mL polymer as the optimal concentration. Changes in the electrode microstructure and capacitive behavior were observed through polymer modification. The peak potential shifted from 0.2 V to approximately 0.15 V. It is known in the literature that peak potentials can vary with surface modification.^62–64^

#### 3.3.5 Analytical Performance of VEA-imp-poly­(HMA-*co*-PA)/SPCE

Under the obtained optimal conditions, the analytical performance of VEA-imp-poly­(HMA-*co*-PA)/SPCE system was elucidated in the presence of different VEA concentrations. To perform analytical analyses, six different samples were prepared with VEA concentrations of 0.1, 0.25, 0.375, 0.5, 0.75, and 1.0 mg/mL. The obtained DPV current values were statistically calculated, and limit of detection (LoD) (3.3× S/N) and limit of quantification (LoQ) (10× S/N) values were statistically determined from Figure 9. Under the defined optimal conditions, LoD value for VEA-imp-poly­(HMA-*co*-PA)/SPCE was found to be 37.1 μg/mL and LoQ value was 112.3 μg/mL (*R* ^2^: 0.9986). The linear working range of the modified sensor extends from 112.3 to 3000 μg/mL.

**Figure 9:**
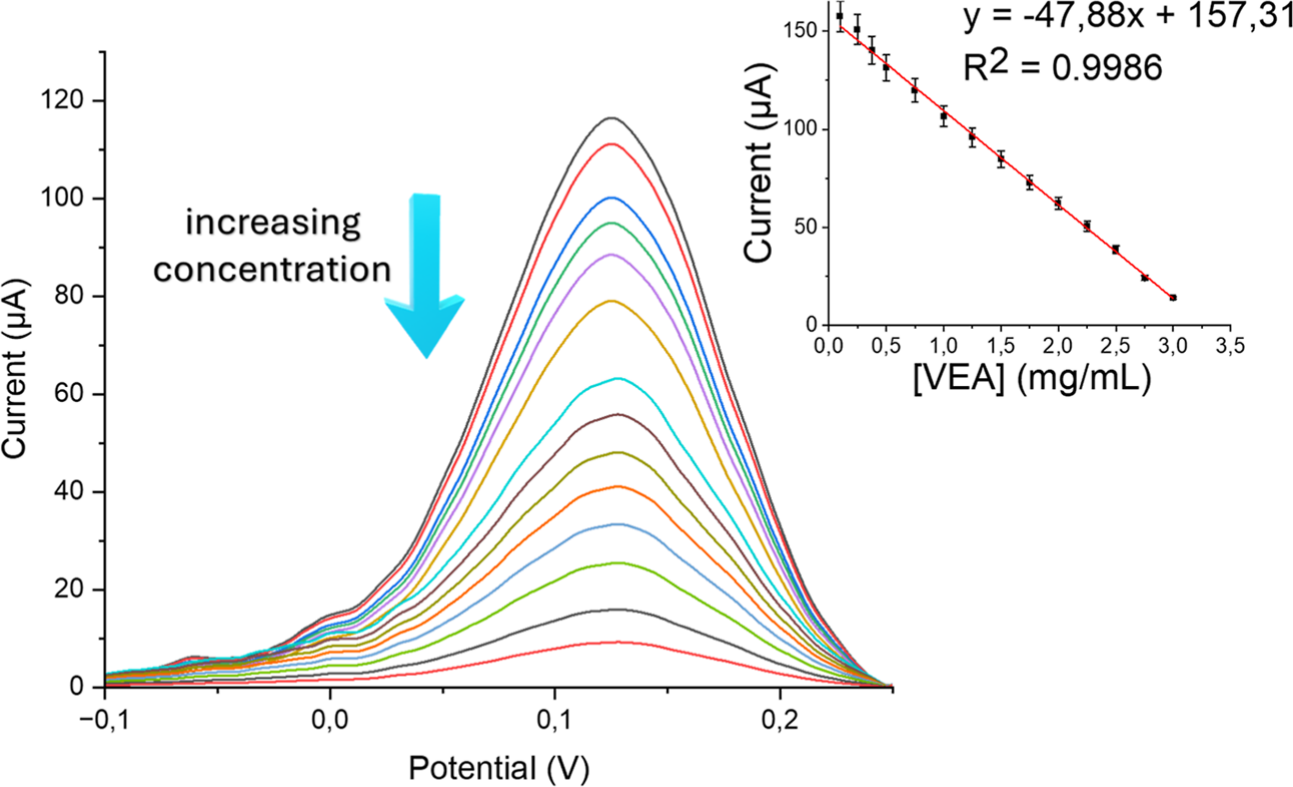
DPV responses for increasing VEA concentration in 10 mM Fe­(CN)_6_^3–/4–^, scanned over the potential range −0.1 ≤ *E* ≤ 0.3 V.

In studies examining lung injuries (EVALI) associated with e-cigarette use, the concentrations of VEA typically range from 0.1 mg/mL to 10 mg/mL.^6^ In this context, the VEA-imp-poly­(HMA-*co*-PA)/Nafion/SPCE sensor system demonstrates a LoQ value very close to the lowest concentration associated with EVALI, 0.1 mg/mL, indicating that the system can detect even the lowest concentrations linked to EVALI symptoms. Additionally, the modified sensor system operates with high sensitivity at concentrations as high as 3 mg/mL, further supporting the potential use of this system in EVALI-related cases.

According to Table 1, the p­(HMA-*co*-PA)/SPCE sensor platform developed in this study offers a broader linear range compared to similar VEA detection systems in the literature. Our sensor provides a wide linear range from 112.3 μg/mL to 3.0 mg/mL. This feature presents a significant advantage compared to previous studies and demonstrates that our sensor exhibits high performance over a wider concentration range. Another important contribution of this study is that the sensor can effectively operate in complex matrices such as electronic cigarette liquids. Our newly developed sensor platform is designed to accommodate a broader spectrum of applications. In conclusion, this study introduces an innovative sensor platform for the electrochemical detection of vitamin E acetate, making a valuable contribution to the literature. Notably, its capability to provide reliable analysis in challenging matrices enhances its potential for practical applications.

**Table 1:** Comparison of This Study with Previously Reported Voltametric VEA Sensors (DPV: Differential Pulse Voltammetry and SWV: Square Wave Voltammetry)

electrode	technique	linear range (μg/mL)	LoD (μg/mL)	references
p(HMA-*co*-PA)/SPCE	DPV	∼112.3 to 3000	37.1	this study
SO/GCPE	SWV	∼0.24 to ∼18.91	∼0.047	^65^
Au/Pan/γ-Al_2_O_3_	DPV	∼0.038 to ∼3.07	∼0.03	^66^
SPGNE	SWV	∼0.08 to ∼5	∼0.012	^67^
platinum microelectrode	SWV	∼25.83 to ∼905.2	∼8.61	^68^
CPE/SDS	SWV	∼43.06 to ∼516.68	∼15,9	^69^

### 3.4 Determination of VEA in E-Cigarette Liquid

To assess the performance of the VEA-imp-poly­(HMA-*co*-PA)/SPCE sensor in real e-cigarette liquid samples (containing nicotine, propylene glycol, and vegetable glycerin), VEA was added to commercially purchased e-cigarette liquids using the standard addition method. Due to the high viscosity and diffusion limitations of e-cigarette liquids, direct measurement on the sensor resulted in unstable signals. To mitigate these issues, a 20-fold dilution was applied using a Fe­(CN)_6_^3–/4–^ solution, which provided stable current responses.

The diluted e-cigarette liquid samples were analyzed, and the current values obtained were compared with those from a standard PBS solution to evaluate matrix effects and assess the sensor’s performance in the presence of potential interferences. The results were expressed as percent recovery, allowing for a quantitative assessment of sensor accuracy and reliability in complex e-cigarette liquid matrices (Table 2).

**Table 2:** % Recovery Values for Different Concentrations (1 mg/mL, 2 mg/mL, and 3 mg/mL) of VEA in Electronic Cigarette Liquid after Applying a 20-Fold Dilution With Fe­(CN)_6_^3–/4–^Solution

VEA (mg/mL) in e-cigarette liquid	% recovery
1	96.83 ± 2.79
2	101.16 ± 3.84
3	102.56 ± 2.67

As a result of VEA determination experiments in electronic cigarette liquid samples, recovery values ranging from 96.83% to 102.56% were obtained for three selected concentrations within the linear working range. The 20-fold dilution played a crucial role in obtaining stable current values, effectively addressing the viscosity issue and enabling the acquisition of stable signals. These results indicate that the VEA-imp-poly­(HMA-*co*-PA)/SPCE sensor system operates with high selectivity and stability in liquid samples. Thus, it is understood that the designed sensor system has strong potential for use in real sample analyses.

### 3.5 Machine Learning to Improve Sensitivity

To evaluate the effectiveness of machine learning algorithms in classifying VEA-positive cases, five supervised learning models were implemented, trained, and tested. These models include Logistic Regression, SVM with an RBF kernel, Random Forest, XGBoost, and NNs. Each model was evaluated based on critical metrics, such as accuracy, precision, recall, and F1-score.

Logistic Regression served as a baseline model due to its simplicity and interpretability. It is a linear model that predicts the probability of a binary outcome using a logistic function. Despite its computational efficiency, Logistic Regression struggled to capture the nonlinear relationships inherent in the data, resulting in moderate performance metrics. The model achieved an accuracy of 63%, with a recall of 62% and an F1-score of 62%, indicating its limited capacity to handle the complexity of the data set. The lower accuracy and F1-score observed in logistic regression may be attributed to the nonlinear nature of the sensor measurements, which this model is inherently less equipped to capture. The confusion matrix highlights its limitations in handling the nonlinear complexity of the data set, as it frequently misclassifies positive and negative cases as shown in Figure 10a.

**Figure 10:**
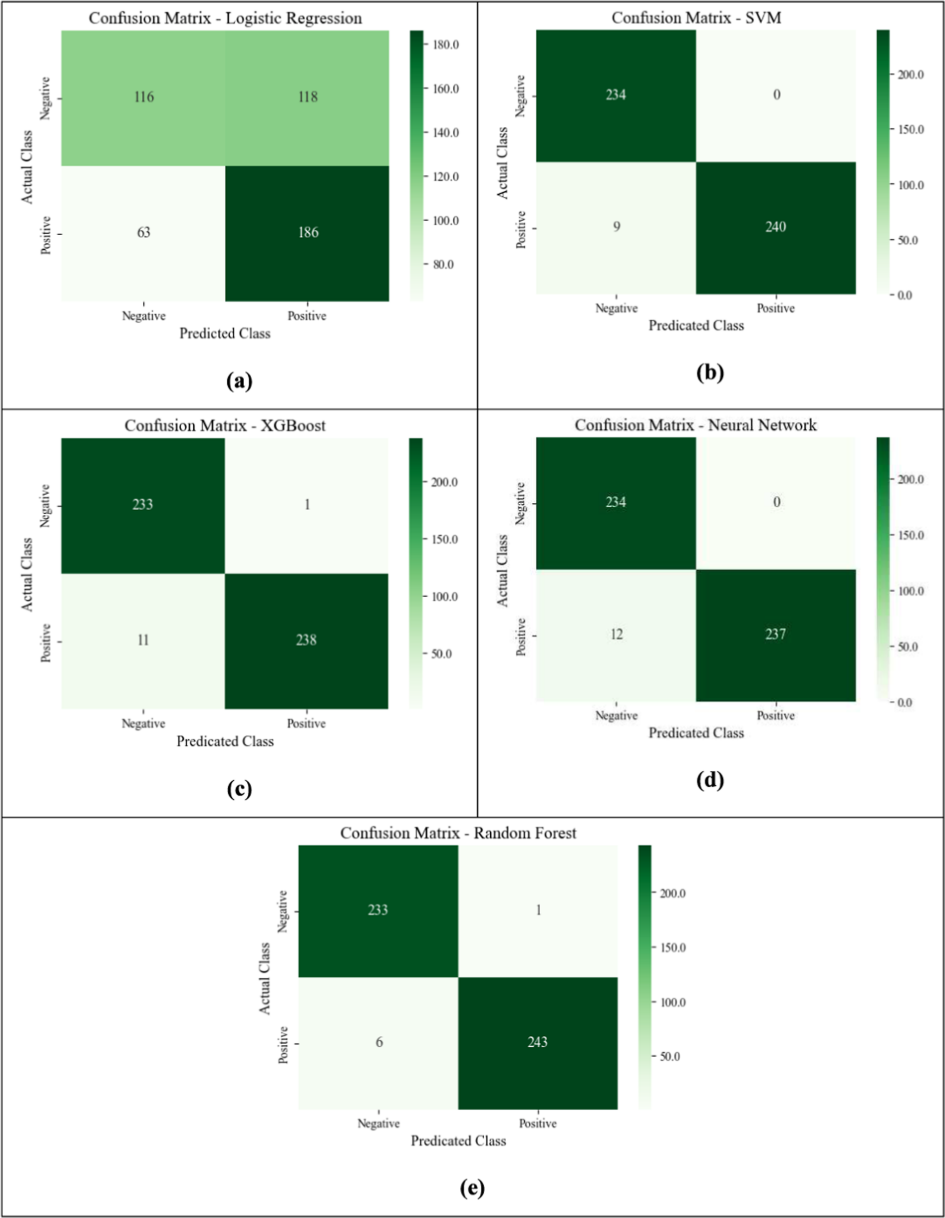
Confusion matrix for ML models. (a) Logistic Regression (b) SVM-RBF Kernel (c) XGBoost (d) NNs (e) Random Forest.

SVM, equipped with a RBF kernel, demonstrated superior performance compared to Logistic Regression. By transforming the input data into a higher-dimensional space, SVM effectively handled the nonlinear decision boundaries present in the data set. The model achieved an accuracy of 98%, with a recall of 98% and an F1-score of 98%. These results highlight the strength of SVM in classifying complex data sets with well-separated classes. The confusion matrix Figure 10b shows minimal false negatives and false positives, emphasizing its robustness in classifying VEA-positive cases.

Random Forest, an ensemble learning algorithm, emerged as one of the top-performing models in this study. By constructing multiple decision trees and aggregating their outputs, Random Forest achieved high robustness and generalization capabilities. It attained an accuracy of 99%, a recall of 99%, and an F1-score of 99%. Furthermore, its feature importance analysis provided valuable insights into the most influential factors contributing to VEA-positive classification, making it an interpretable and reliable choice. As illustrated in Figure 10e, its confusion matrix highlights its near-perfect classification performance, with negligible false negatives and false positives. The model’s feature importance analysis further emphasized the key drivers for accurate predictions.

XGBoost, a scalable and efficient gradient-boosting algorithm, matched Random Forest in performance while offering faster training times. The model achieved an accuracy of 98%, a recall of 98%, and an F1-score of 98%. Its ability to handle imbalanced data sets and provide probabilistic predictions further solidified its suitability for the classification task. Additionally, XGBoost’s hyperparameter tuning capabilities allowed for enhanced model performance without significant computational overhead. Despite achieving comparable classification accuracy to XGBoost, as shown in Figure 10c, Random Forest exhibited superior performance, particularly with respect to F1-score and precision

NNs, designed to learn complex, nonlinear relationships, also performed strongly. Composed of multiple layers of interconnected neurons, the model achieved an accuracy of 98%, a recall of 98%, and an F1-score of 98%. While NNs demonstrated competitive performance, they required more computational resources for training and exhibited slightly lower precision compared to Random Forest and XGBoost. Nonetheless, their flexibility and scalability make them a viable option for similar classification tasks. While its confusion matrix as illustrated in Figure 10d indicates high recall and overall performance, it slightly underperformed compared to Random Forest and XGBoost in precision, making it less suitable for applications where false positives must be minimized.

Table 3 presents the performance metrics of five machine learning models—Logistic Regression, SVM with RBF Kernel, Random Forest, XGBoost, and NNs—evaluated for their ability to classify VEA. The metrics used for evaluation include precision, recall, F1-score, and accuracy, providing a comprehensive understanding of each model’s classification capabilities. Random Forest outperformed other models in all metrics, making it the most reliable choice for this classification task. Models like SVM and XGBoost balanced precision and recall effectively, while NNs slightly favored recall over precision. As a linear model, Logistic Regression was unable to model the complexity of the data set, leading to significantly lower performance compared to other models.

**Table 3:** Performance Metrics of ML Models

model	precision	recall	F1-score	accuracy
Logistic Regression	0.63	0.62	0.62	0.63
SVM (RBF Kernel)	0.98	0.98	0.98	0.98
Random Forest	0.99	0.99	0.99	0.99
XGBoost	0.98	0.98	0.98	0.98
NNs	0.96	0.98	0.98	0.98

The feature importance analysis derived from the Random Forest model highlights the relative contribution of each feature—potential (V) and current (μA)—to the classification of VEA. The computed weights indicate that current (μA) has a higher relative importance compared to potential (V). Specifically, current (μA) accounts for a larger percentage of the model’s decision-making process, which aligns well with the underlying principles of the sensor’s operation.

The residuals distribution of the Random Forest model, as depicted in Figure 11, illustrates its strong predictive performance and reliability. The residuals are centered around zero, indicating that the model’s predictions are unbiased on average and closely align with the actual values. The narrow spread of residuals suggests that the model makes consistent predictions with minimal errors, further supported by the absence of extreme outliers. Additionally, the approximate symmetry of the distribution, resembling a normal curve, demonstrates that the model generalizes well and does not overfit to the training data. The high frequency of residuals near zero highlights the model’s ability to accurately predict most instances, while the low frequency of larger residuals confirms that significant prediction errors are rare. Overall, this analysis underscores the robustness of the Random Forest model in capturing the underlying relationships in the data and its capability to make reliable classifications.

**Figure 11:**
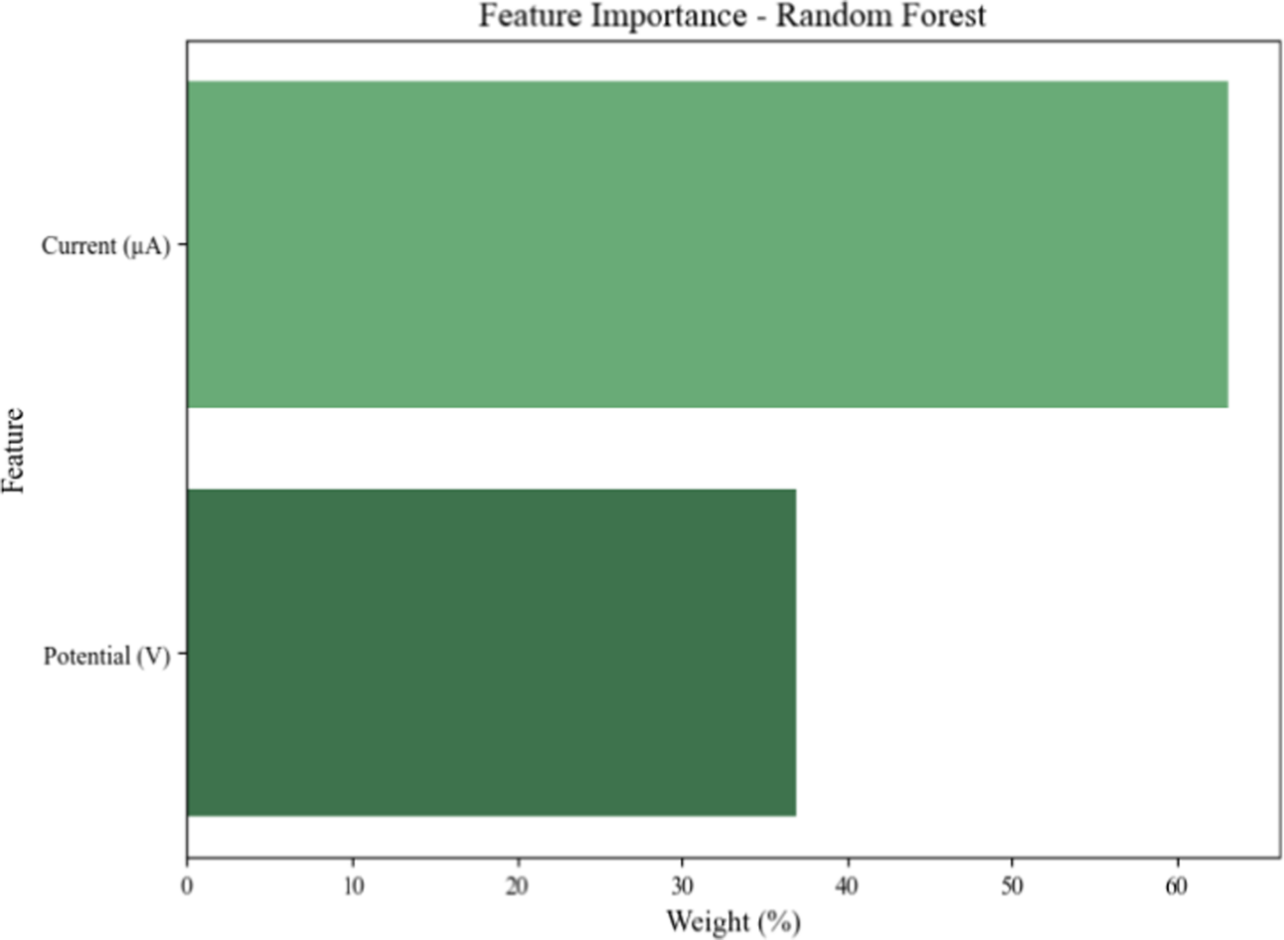
Feature importance for Random Forest model.

These findings highlight the critical role of integrating machine learning models with advanced sensor technologies, particularly to address challenges associated with detecting analytes at low concentrations, thereby enhancing the overall sensitivity and reliability of the sensing system (Figure 12).

**Figure 12:**
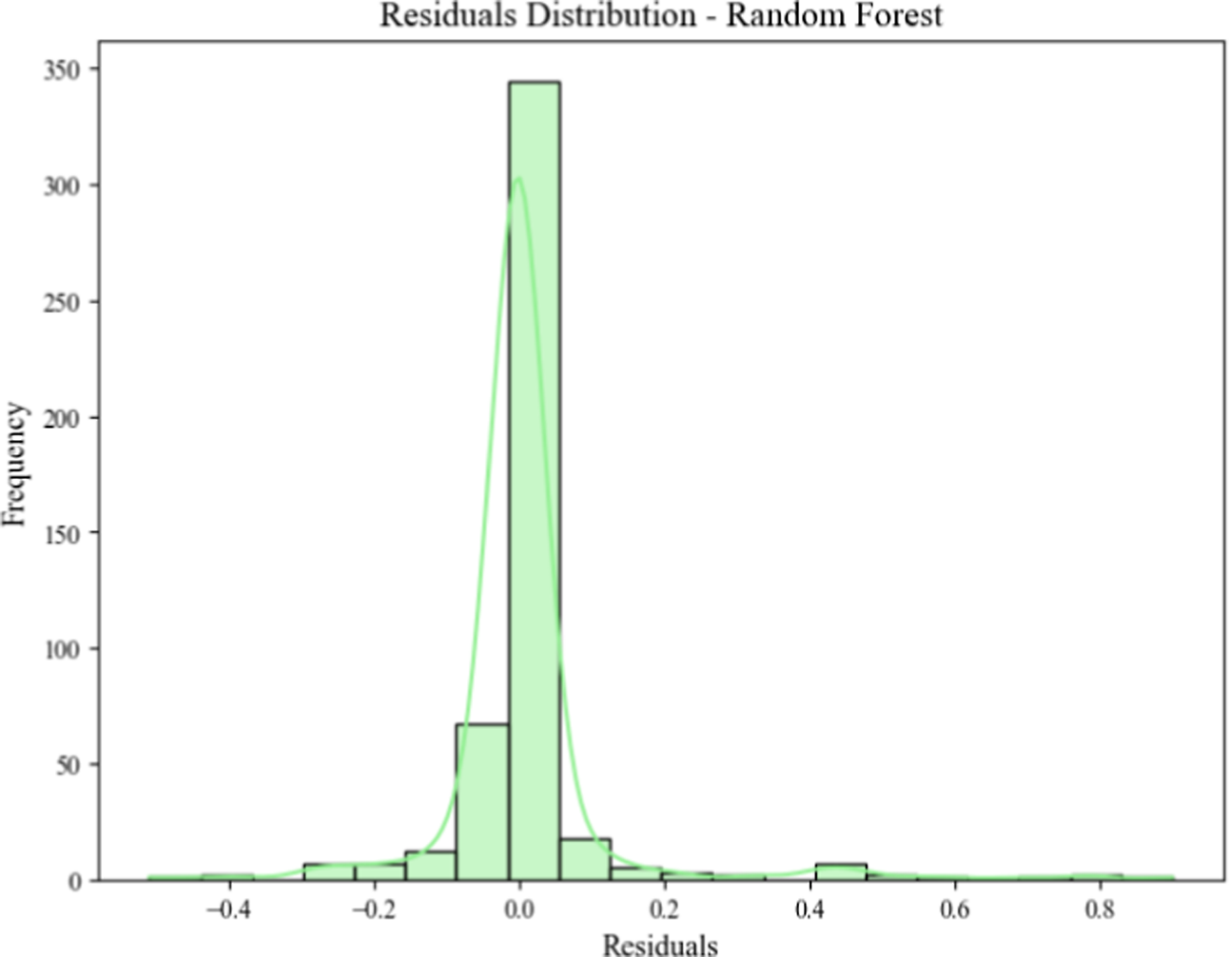
Residuals distribution of Random Forest model.

## 4 Conclusion and Future Work

This study successfully demonstrated the development of a novel MIECS integrated with ML algorithms for the sensitive and selective detection of VEA in e-cigarette liquids, addressing a critical need in public health monitoring. The sensor, fabricated using VEA-imprinted poly-(HMA-*co*-PA)/Nafion on SPCE, exhibited exceptional analytical performance with a LoQ of 112.3 μg/mL and a wide linear detection range extending to 3.0 mg/mL. Rigorous validation with authentic e-cigarette liquid samples demonstrated high accuracy and selectivity, with recovery rates ranging from 96.83% to 102.56%, confirming the sensor’s robustness in complex matrices.

Despite the sensor’s strong performance, limitations were encountered in detecting VEA at concentrations below the LoQ. To address this challenge, machine learning algorithms, specifically the Random Forest algorithm, were effectively integrated into the sensing system. The ML model demonstrated exceptional performance, achieving an accuracy of 99% along with high precision, recall, and F1-scores, enabling reliable classification of VEA presence even at subdetection limit concentrations. This integration significantly enhanced the analytical capabilities of the sensor system, minimizing the risk of false negatives—a critical factor for accurate and reliable health risk assessments.

Moreover, the integration of ML models into the sensing workflow enabled the reliable identification of VEA even in electrochemical measurements with ambiguous or subthreshold signal profiles. By learning from the voltage–current response patterns, the models effectively classified the presence of VEA with minimal false negatives, thereby serving as a crucial interpretive layer within the analytical process and strengthening the validity of the detection outcome.

Future research will focus on translating this promising technology into a practical and user-friendly platform for real-time VEA detection. This involves the development of a miniaturized and portable device compatible with mobile platforms, enabling rapid, on-site analysis of e-cigarette liquids. Leveraging the computational power of mobile devices for on-device ML-driven data processing will enhance the accessibility and practicality of this technology. Furthermore, future studies will explore the versatility of this hybrid MIECS-ML approach by extending its application to the detection of other harmful compounds present in e-cigarette liquids and other complex matrices. This will involve optimizing the sensor’s molecular imprinting process for various target analytes and exploring advanced ML techniques, such as deep learning, to further enhance detection accuracy and address more complex analytical challenges. This research exemplifies the transformative potential of integrating AI with advanced sensor technologies for addressing critical public health concerns and advancing the field of environmental and biomedical monitoring.

## Supplementary Material



## Notes

Zeta potential analysis of VEA-imp-poly­(HMA-*co*-PA) nanoparticles (Figure S1); dynamic light scattering-based size distribution of the synthesized nanoparticles (Figure S2); UV–vis calibration curve for quantitative determination of VEA (Figure S3) (PDF)
