# Supplementary material for: Harnessing Machine Learning to Revolutionize Electrochemical Detection of Vitamin E Acetate in E‑Liquids
Source: ACS Omega. 2025 Jun 13;10(25):27098–111. doi: 10.1021/acsomega.5c02363 (PMC12223865; doi:10.1021/acsomega.5c02363)
Supplement: Supplementary file 1 [file ao5c02363_si_001.pdf]

Supporting Information

Harnessing Machine Learning to Revolutionize Electrochemical Detection of Vitamin E Acetate in E-Liquids

Emine Sezer[1]*, Emre Dokuzparmak[2*], Hilal Özçelik[3], Esra Yaşar[3], Tarık Kaya[2], Timuçin Güner[3], Sinan Akgöl[3,4]

[1] Department of Computer Engineering, Faculty of Computer and Information Science, Ege University, Bornova, Izmir 35100, Turkey

[2] Department of Bioengineering, Faculty of Engineering, Ege University, Bornova, Izmir 35100, Turkey

[3] Department of Biochemistry, Faculty of Science, Ege University, Bornova, Izmir 35100, Turkey

[4] Sabancı University Nanotechnology Research and Application Center (SUNUM), Tuzla, Istanbul 34956, Turkey

*e-mail : emine.sezer@ege.edu.tr

1. Zeta Size and Potential Analysis

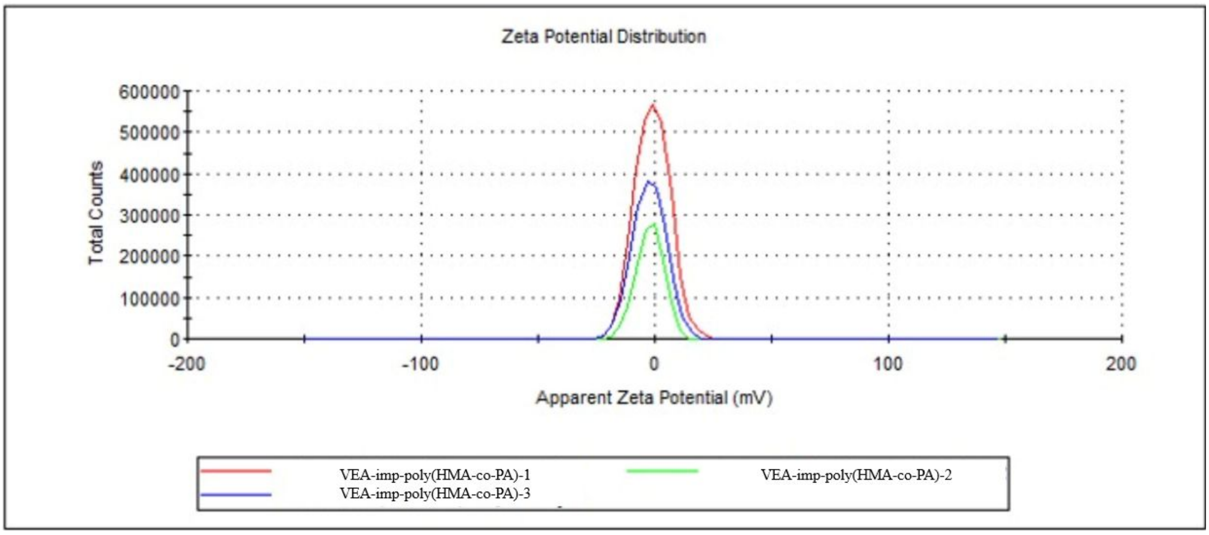

Figure S1. Zeta potential of the VEA-imp-poly(HMA-co-PA).

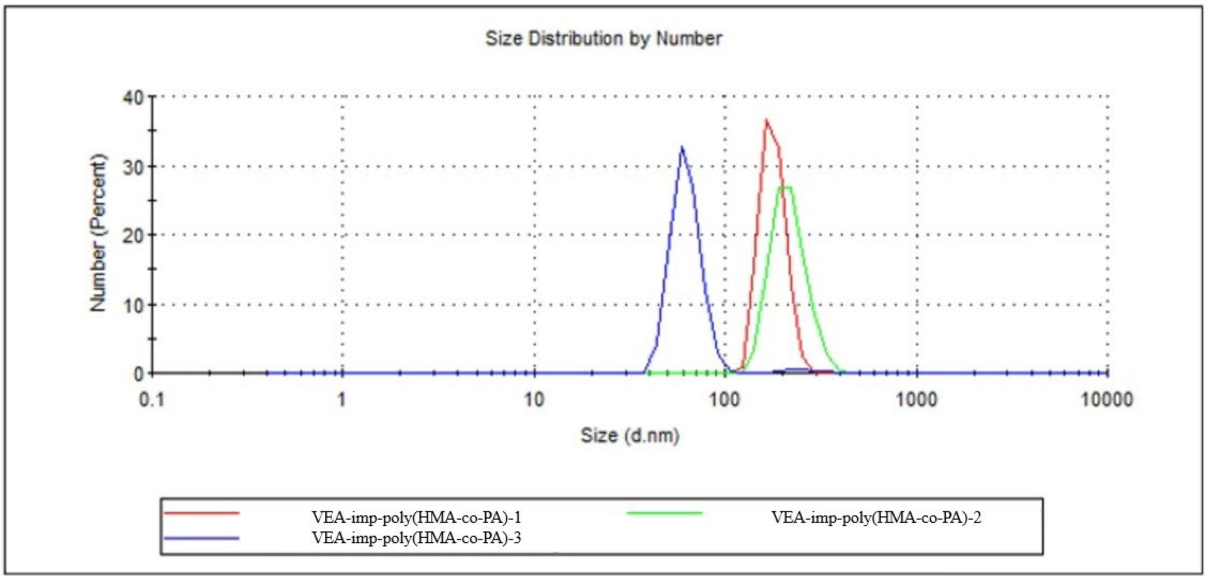

Figure S2. Zeta size of the VEA-imp-poly(HMA-co-PA).

2. Calibration Graphic

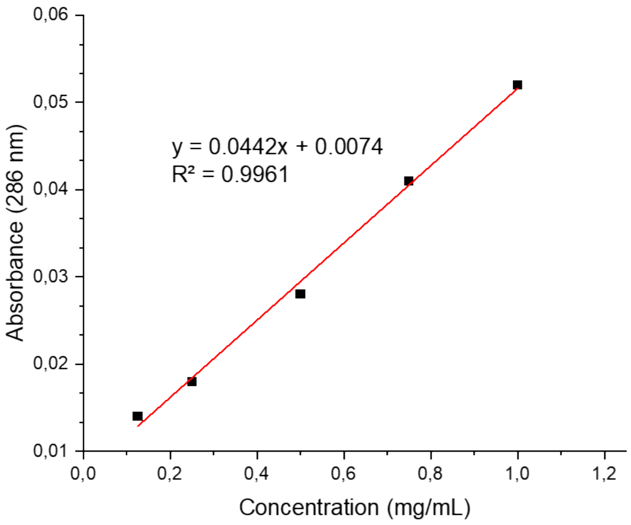

Figure S3. Calibration graphic of VEA
